# AttributeRank: An Algorithm for Attribute Ranking in Clinical Variable Selection

**DOI:** 10.1111/jep.14257

**Published:** 2024-12-20

**Authors:** Donald Douglas Atsa'am, Ruth Wario, Pakiso Khomokhoana

**Affiliations:** ^1^ Department of Computer Science, College of Physical Sciences Joseph Sarwuan Tarka University Makurdi Benue State Nigeria; ^2^ Department of Computer Science and Informatics, Faculty of Natural and Agricultural Sciences University of the Free State ‐ QwaQwa Campus Phuthaditjhaba Free State South Africa; ^3^ Department of Computer Science and Informatics, Faculty of Natural and Agricultural Sciences University of the Free State Bloemfontein Free State South Africa

**Keywords:** attribute ranking, AttributeRank, clinical classification, risk difference, variable selection

## Abstract

**Background:**

Risk difference is a valuable measure of association in epidemiology and healthcare which has the potential to be used in medical and clinical variable selection.

**Objective:**

In this study, an attribute ranking algorithm, called AttributeRank, was developed to facilitate variable selection from clinical data sets.

**Methods:**

The algorithm computes the risk difference between a predictor and the response variable to determine the level of importance of a predictor. The performance of the algorithm was compared with some existing variable selection algorithms using five clinical data sets on neonatal birthweight, bacterial survival after treatment, myocardial infarction, breast cancer, and diabetes.

**Results:**

The variable subsets selected by AttributeRank yielded the highest average classification accuracy across the data sets, compared to Fisher score, Pearson's correlation, variable importance function, and Chi‐Square.

**Conclusion:**

AttributeRank proved to be more valuable in attribute ranking of clinical data sets compared to the existing algorithms and should be implemented in a user‐friendly application in future research.

## 1 Introduction

In modelling, the concept of variable selection refers to the process of choosing a subset from the domain of input variables that result in models with good fit and high accuracy. It has been pointed out that not all the variables that make up the data set of a problem domain convey information of interest and relevance in modelling [1]. When irrelevant variables are included in a predictive model, some metrics of interest such as the Alkaike Information Criteria (AIC), variance, and degrees of freedom are unnecessarily inflated which have negative effects on model performance [2, 3, 4]. Variable selection is necessary to guard against the inclusion of less important variables in models. A good variable selection algorithm should be capable of producing a reduced number of variables that result in a model with best fit and high predictive accuracy [5]. It is equally important that variable selection algorithms are easy to implement, with minimal overhead costs such as space and memory requirements [6].

There are basically three methodologies of variable selection including filter methods, wrapper methods, and embedded methods [7, 8]. According to Kaushik [8], variable selection under the filter methods is a form of preprocessing that does not depend on any learning algorithm. Rather, this method depends on the values obtained with respect to the correlation of each independent variable with the response variable. Correlation measures such as Pearson's correlation, ANOVA, and Chi‐square are applied in the filter methods. The wrapper methods use subsets of variables to train and test models iteratively for accuracy [9]. Based on the accuracy of the previous model, variables are either added or removed from the subset until a subset that produces a model with a reasonable accuracy is obtained. Wrapper methods are computationally intensive when the number of variables is large and consequently several subsets to evaluate exist. Embedded methods combine variable selection and model building into the same algorithm [7]. Some examples include the Ridge and Lasso regressions which have in‐built penalisation procedures that reduce overfitting inherent in the machine learning algorithms [8].

In recent years, there has been a surge in the number of emerging diseases across the world such as the corona virus disease 2019 (COVID‐19), avian influenza, Crimean‐Congo haemorrhagic fever, and Lassa fever [10, 11]. According to WHO [12], some of the factors influencing the surfacing of emerging diseases include changes in farming practice and the environment, changes in animal and human demography, and pathogen changes. If these factors continue to exist, there will highly likely be some instances of emerging diseases across the globe. Similarly, there will continue to be scientific responses to model and tackle the situations as they emerge. In most cases, part of the solution domain to handle a disease outbreak requires the selection of important variable subset for modelling. Though there are several filter variable selection algorithms such as the Fisher score, Pearson's correlation, and Chi‐square, these algorithms were not designed specifically to model disease situations. Based on this background, the objective of this study was to develop a filter algorithm, named AttributeRank, which ranks the variables of clinical, epidemiological, and medical data sets. The ranking criterion is anchored on a statistical quantity known as the risk difference, which is a measure of the risk of a disease' outcome in the exposed group that is attributable to the exposure [13]. It is expected to be a useful tool for researchers and practitioners modelling with health‐related data sets. AttributeRank eliminates the time often spent by medical, public health, and clinical practitioners or researchers in deciding which of the pre‐existing general‐purpose filter methods to use when modelling a disease.

## 2 Related Literature

This section presents some existing variable selection algorithms relevant to this study. Furthermore, the risk difference, a statistical measure deployed for the design of the new attribute ranking algorithm, is defined in this section.

### 2.1 Some Existing Variable Selection Algorithms

#### 2.1.1 Fisher Score

Fisher score evaluates the importance of the i‐th independent variable of a data set using Equation (1):

(1) $$F_{i}=|\frac{\bar{X_{1}}(i)-\bar{X_{0}}(i)}{d_{1}^{2}(i)+d_{0}^{2}(i)}|$$

where ${\bar{X}}_{1}(i)$ and $d_{1}(i)$ are respectively the mean and the standard deviation of the i‐th feature with class 1; ${\bar{X}}_{0}(i)$ and $d_{0}(i)$ are respectively the mean and the standard deviation of the i‐th feature with class 0 [5, 14].

#### 2.1.2 Pearson's Correlation

Pearson's correlation computes the importance of an attribute as in Equation (2) [15].

(2) $$P_{i}=\frac{\mathrm{Cov}(X_{i},Y)}{\sqrt{\mathrm{Var}(X_{i})\times \mathrm{Var}(Y)}}$$

where $X_{i}$ and Y are respectively the i‐th variable and the outcome label, Cov() and Var() are the covariance and variance, respectively.

#### 2.1.3 VarImp Function

The R programming language consists of a function known as varImp (variable importance) within the caret package [16], which serves the purpose of attribute ranking. With a logistic regression model, the varImp function determines attribute importance by computing the t‐statistic for each predictor. The t‐statistic is evaluated as shown in Equation (3) [5, 17].

(3) $$t_{i}=\frac{|\bar{X_{1}}(i)-\bar{X_{0}}(i)|}{\sqrt{\frac{d_{1}^{2}(i)}{n_{1}}+\frac{d_{0}^{2}(i)}{n_{0}}}}$$

where $d_{1},d_{0},{\bar{X}}_{1},{\bar{X}}_{0}$ are same as defined in Equation (1), $n_{0}$ and $n_{1}$ are respectively the total records in the class 0 and 1.

#### 2.1.4 Chi‐Square

Chi‐square is a measure of the level of dependence of a predictor variable on the outcome variable. It measures how the observed count, O, and the expected count, E, of the data of two variables deviate from each other. It is computed as shown in Equation (4) [18].

(4) $$\chi_{c}^{2}=\sum \frac{{\left(O_{i}-E_{i}\right)}^{2}}{E_{i}}$$

where c is the degrees of freedom, O is the observed value(s), and E is the expected value(s). In attribute selection, features that have higher values of chi‐square are selected for modelling.

#### 2.1.5 Variable Selection With Odds Ratio

Atsa'am [19] designed a feature selection algorithm that depends on odds ratio to evaluate the importance of each feature in a data set. The algorithm is summarised in Equation (5).

(5) $${\mathrm{IMP}}_{j}=\left(\frac{\sum_{i=1,j=1}^{m,n}Eij\times \sum_{i=1,j=1}^{m,n}Fij}{\sum_{i=1,j=1}^{m,n}Gij\times \sum_{i=1,j=1}^{m,n}Hij}\right)$$

where, ${\mathrm{IMP}}_{j}$ is the importance score of the jth predictor (j=1,…,n), Eij = total data instances with input = 0 and output = 0, Fij = total data instances with input = 1 and output = 1, Gij = total data instances with input = 0 and output = 1, and Hij = total data instances with input = 1 and output = 0.

#### 2.1.6 Feature Selection With Risk Ratio

In a relatively recent study, Bodur and Atsa'am [5] developed a feature selection algorithm with risk ratio. This is a variable ranking algorithm that uses risk ratios to score predictor variables in modelling. The formula for the algorithm is given in Equation (6)

(6) $$I_{j}=\left(\frac{\sum_{i=1,j=1}^{m,n}Wij}{\sum_{i=1,j=1}^{m,n}Wij+\sum_{i=1,j=1}^{m,n}Xij}\right)\;.\;\left(\frac{\sum_{i=1,j=1}^{m,n}Yij+\sum_{i=1,j=1}^{m,n}Zij}{\sum_{i=1,j=1}^{m,n}Yij}\right)$$

where $I_{j}$ is the importance score of the *j*th variable, $W_{ij}$ is the total number of observations with input = 1 and output = 1, $X_{ij}$ is the total number of observations with input = 1 and output = 0, $Y_{ij}$ is the total number of observations with input = 0 and output = 1, $Z_{ij}$ is the total number of observations with input = 0 and output = 0, *m* is the number of observations in the data set, and *n* is the number of predictor variables. For every predictor, the algorithm separately sums up the total number of observations in each of the four categories and evaluates the importance score of each predictor.

### 2.2 Risk Difference: A Measure Employed for the Proposed Algorithm

Kim [13] defines risk difference (RD) by Equation (7). This definition is based on the assumption that for a binary independent attribute X and a binary outcome variable Y, let $a_{11}$= total observations where X=1 and Y=1, $b_{10}$ = total observations where X=1 and Y=0, $c_{01}$= total observations where X=0 and Y=1, and $d_{00}$ = total observations where X=0 and Y=0.

(7) $$(7)RD=\left(\frac{a_{11}}{a_{11}+b_{10}}\right)-\left(\frac{c_{01}}{c_{01}+d_{00}}\right)$$

RD can evaluate to either a negative or a positive number and is a measure used in epidemiology to quantify the risk of a disease outcome in the exposed group that is attributable to the exposure.

## 3 Materials and Methods

In this section, the methods and procedure of how the proposed algorithm, AttributeRank, was developed are presented. Furthermore, insights into the experiments that were conducted to compare the performance of the new algorithm with four existing algorithms, namely, Fisher score, Pearson's correlation, Chi square, and varImp are provided.

### 3.1 Design of AttributeRank Algorithm

The measure adopted in AttributeRank for ranking attributes is the risk difference. As noted, risk difference has been used extensively in epidemiology to quantify the risk of a disease' outcome in the exposed group that is attributable to the exposure. The underlying mechanism of the proposed algorithm is to compute the risk difference between each predictor variable and a clinical outcome from the data set. The value of the risk difference literally quantifies the extent to which the predictor is important in modelling the outcome.

The algorithm consists of three main modules: (i) the module for dichotomising the data set, (ii) the module for evaluation of the four quantities (a_11_, b_10_, c_01_, and d_00_) required for computation of risk difference, (iii) the module for computation of risk difference for each independent‐dependent variable and then rank the variables based on risk difference values. Each of the modules is designed in this section.

- Module one: This module is responsible for transforming all input data to binary format since risk difference is computed on binary data. Using a threshold of 0.4, each data point of the normalised data which is below this value is converted to 0, and 1 otherwise. The pseudocodes for this module appear on lines 4−17 of the algorithm in the Appendix.
- Module two: In this module, the four quantities (a_11_, b_10_, c_01_, and d_00_) required for computation of risk difference are evaluated from the binarized data generated in module one. These quantities are defined in Equation (7). Specifically, the statements on lines 31 and 32 count the total number of occurrences where input = 1 and output = 1; lines 34 and 35 count the total number of occurrences of input = 1 and output = 0; lines 37 and 38 count all observations where input = 0 and output = 1; and lines 40 and 41 count all occurrences where input = 0 and output = 0 (see the Appendix).
- Module three: In this module, the four quantities obtained in module two are employed to compute the risk difference for each data attribute. Effectively, the value obtained for each data attribute, termed AttributeRank, serves as the score of how important the attribute is in modelling a clinical outcome. Specifically, the statements on lines 55 to 61 implement the RD formula given in Equation (7) and then return the absolute value of the result—see the Appendix.

It is important to emphasise that AttributeRank requires a data set that has been scaled to the interval [0,1]. A commonly used method of normalising a data set to this format is the min‐max normalisation [19]. The min−max normalisation scales down all data points to run in the interval 0 and 1 so that extreme values do not adversely affect smaller (or larger) values of the data. Furthermore, the variables of a clinical data come in different units of measurement such as mmHg, mg/dL, and years. Therefore, it is important that these diverse units are normalised to the same scale before deployment of AttributeRank.

Two symbols, namely, # and // were used to provide comments and/or insert empty lines within the algorithm to enhance legibility. Algorithm comments ensure that readers with little programming skills are carried along with respect to what each module, subsection, or procedure of the algorithm seeks to achieve [20]. The algorithm is syntax‐generic and can be implemented in any appropriate programming language. Equation (8) gives a summary of AttributeRank.

(8) $$AttributeRank_{j}=|\left(\frac{\sum_{i=1,j=1}^{m,n}a_{ij}}{\sum_{i=1,j=1}^{m,n}a_{ij}+\sum_{i=1,j=1}^{m,n}b_{ij}}\right)-\left(\frac{\sum_{i=1,j=1}^{m,n}c_{ij}}{\sum_{i=1,j=1}^{m,n}c_{ij}+\sum_{i=1,j=1}^{m,n}d_{ij}}\right)|$$

where $AttributeRank_{j}$ is the value of the importance of the jth attribute, j=1,…,n, $a_{ij}$ is the total number of observations with *input* = 1 and *output* = 1, $b_{ij}$ is the total number of observations with *input* = 1 and *output* = 0, $c_{ij}$ is the total number of observations with *input* = 0 and *output* = 1, and $d_{ij}$ is the total number of observations with *input* = 0 and *output* = 0.

### 3.2 Experimental Data sets

The following clinical data sets were used to test the performance of AttributeRank, compared to Fisher score, Pearson's correlation, Chi‐square, and varImp. The data sets were obtained from Rdatasets [21] at [https://vincentarelbundock.github.io/Rdatasets/articles/data.html]. They are freely available for research purposes and there were no requirements for prior ethics approval.

- Low infant birthweight (Birthweight): The birthweight data consist of 189 observations and eight independent variables, such as mother's age and mother's weight, which are the risk factors associated with low infant birthweight. The data have a binary outcome variable to indicate low or normal birthweight. The data are freely available from the R package, MASS [22, 23].
- Presence of bacteria after drug treatments (Bacteria): These data consist of 220 records related to whether the bacteria, H. influenzae, are present or not after a course of treatment for children with otitis media. The data have four independent variables including active/placebo and compliance, with a binary response variable that indicates the presence or absence of the bacteria [23].
- Females suffering from myocardial infarction (Myocardial): The data consist of 1295 records and nine independent variables such as high cholesterol and smoking status. The data are about women suffering from myocardial infarction. There is a binary outcome variable to indicate whether a woman died from the disease or not [24].
- Biopsy data on breast cancer patients (Cancer): These are data on 699 patients suffering from breast cancer [23]. They consist of 699 observations and nine independent variables including clump thickness and uniformity of cell size, with a binary outcome variable to tell whether the cancer is benign or malignant.
- Diabetes in Pima Indian women (Diabetes): The data are made up of 200 observations of Indian women tested for diabetes. They have seven explanatory variables which include diastolic blood pressure and glucose concentration, with a binary class variable that indicates whether the woman is diabetic or not [23]. The properties of the experimental data sets are summarised in Table 1.

**Table 1:** Experimental data sets.

Data set	No. of records	No. of independent variables	No. of records in class 0	No. of records in class 1
Birthweight	189	8	130	59
Bacteria	220	4	43	177
Myocardial	1295	9	321	974
Cancer	683	9	444	239
Diabetes	200	7	132	68

### 3.3 Analytical Approach

Before modelling, the data sets were separately preprocessed by first transforming the data points to uniform scales using min‐max normalisation. This effectively converted all data points to fall within the interval [0,1]. After the normalisation, each of the five variable selection algorithms (the pre‐existing four and AttributeRank) were executed on the data sets to rank the attributes in the order of importance. The most important attributes of each data set were then selected for classification modelling. The variable subset sizes selected by each algorithm are shown in Table 2. Each data set was randomly split into two parts made up of 80% and 20% of the observations. The 80% of the observations were employed in fitting classification models with logistic regression, using the subset of important variables specified by each algorithm. On the other hand, the 20% of the observations were reserved for testing the predictive accuracy of the models.

**Table 2:** Variable subset per ranking algorithm.

Data set	AttributeRank	Fisher score	Pearson's correlation	varImp	Chi‐square
Birthweight	4	4	4	4	3
Bacteria	3	2	3	2	2
Myocardial	5	4	3	5	5
Cancer	4	3	3	2	2
Diabetes	3	3	3	3	3

## 4 Results

Table 2 provides the subset sizes specified by each variable selection algorithm in each data set. The algorithms rank variables with numerical values that indicate how important a variable is in modelling. The higher the importance value, the better the variable in modelling.

The performances of the classification models constructed with the variable subsets were evaluated in terms of the goodness of fit and classification accuracy. Goodness of fit was evaluated using the McFadden's pseudo *r*‐squared (*R* ^2^), which produced the results shown in Table 3.

**Table 3:** Goodness of fit evaluation with McFadden's pseudo *R* ^2^.

Data set	AttributeRank	Fisher score	Pearson's correlation	varImp	Chi‐square
Birthweight	0.10	0.13	0.10	0.12	0.12
Bacteria	0.07	0.05	0.04	0.06	0.04
Myocardial	0.06	0.07	0.02	0.04	0.06
Cancer	0.84	0.83	0.83	0.79	0.76
Diabetes	0.30	0.30	0.30	0.30	0.30
Average	0.27	0.28	0.26	0.26	0.26

The McFadden's pseudo *R* ^2^ is a measure of how well a logistic regression model fits the data set. When comparing two or more models, the one with a higher value of pseudo *R* ^2^ provides a better fit. It could be observed from Table 3 that AttributeRank produced an average of 0.27 pseudo *R* ^2^ across the five data sets. This is above the average values produced by the Pearson's correlation, varImp, and the Chi‐square.

Furthermore, the classification accuracies of the models constructed with the various variable subsets were evaluated on the test sets. The results are given in Table 4.

**Table 4:** Classification accuracy (%).

Data set	AttributeRank	Fisher score	Pearson's correlation	varImp	Chi‐square
Birthweight	74.00	68.00	68.00	76.00	68.00
Bacteria	80.00	75.00	84.00	77.00	80.00
Myocardial	76.00	74.00	77.00	74.00	75.00
Cancer	93.40	91.20	91.20	93.40	91.00
Diabetes	75.00	75.00	75.00	73.00	73.00
Average	79.60	76.60	79.00	78.60	77.40

**Figure 1:**
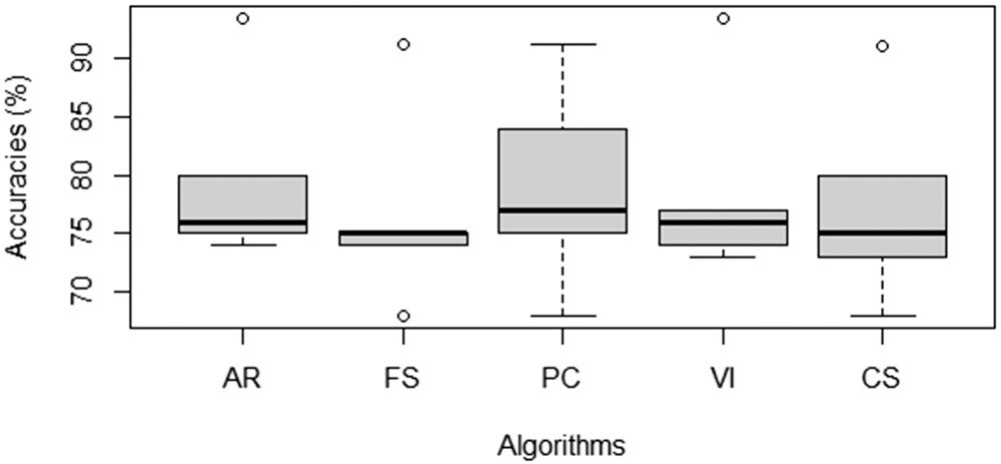
Classification accuracies. AR, AttributeRank; CS, chi‐square; FS, fisher score; PC, Pearson's correlation; VI, varImp.

The classification accuracies in Table 4 can be visualised with the aid of a boxplot shown in Figure 1.

## 5 Discussion

This study was conceived out of the desire to develop a filter variable selection algorithm that performs better than other filter algorithms, especially with health‐related data. This motivated the design of a filter method which uses the statistical measure of association, namely, risk difference to score attributes by their importance. As pointed out by Bodur and Atsa'am [5], the purpose of an attribute selection algorithm is to suggest a subset of variables that will produce models with higher classification accuracy. A viable procedure for deciding which among two or more variable selection methods is the best is to compare the separate models constructed using subsets suggested by each method. This procedure was followed in the present study. The variable subsets suggested by AttributeRank produced an average classification accuracy of 79.60% across the five data sets, surpassing all the other filter methods (see Table 4). The major strength of the new algorithm is in its ability to select variable subsets that produced higher classification accuracies, compared to the existing methods.

In normal clinical or epidemiological evaluations, risk difference can take either a positive or a negative value [13]. When the risk difference is negative, it means there is a decreased risk with the corresponding exposure. In this study however, the algorithm takes the absolute value of the risk difference to eliminate the possibility of a negative value—see line 60 of the algorithm in the Appendix. This means that equal premium is placed on the importance of a variable in predicting the presence or absence of a clinical outcome. This feature is an important quality for a tool meant to be used in clinical modelling where false negatives and false positives are equally crucial.

## 6 Conclusion

There are several filter variable selection algorithms such as the Fisher score, Pearson's correlation, *t*‐statistic, and Chi‐square. However, these algorithms were not designed specifically to model clinical outcomes. Consequently, medical, public health, and clinical practitioners and researchers are often faced with the challenge of deciding which of the pre‐existing filter methods to use when modelling a disease. To resolve this, a filter algorithm named AttributeRank, was designed in the present study through a useful measure in epidemiology and clinical modelling: risk difference. The algorithm ranks each data attribute based on the absolute value of the risk difference score computed from a binarized data set. The performance of AttributeRank was empirically compared with Fisher score, Pearson's correlation, varImp, and Chi‐square. The variable subsets selected by the new algorithm produced a higher average classification accuracy across five clinical data sets, compared to the existing algorithms. Therefore, it is potentially a useful variable selection tool for researchers and practitioners modelling with health‐related data sets. In its present form, the algorithm ranks attributes in the order of importance without specifying a cut‐off point that distinguishes between important and unimportant attributes. It is left to the discretion of the human expert to examine where there is an unusual gap in the sequence of important values to determine the variables to exclude from modelling. In future research, the algorithm should be extended to include procedures and functions that automate the process of determining cut‐off points for important attributes. Furthermore, the algorithm should be implemented as a user‐friendly application with graphical user interfaces for end users with little or no knowledge of algorithms.

## Conflicts of Interest

The authors declare no conflicts of interest.

## Data Availability

The data that support the findings of this study are openly available in [Rdatasets] at [https://vincentarelbundock.github.io/Rdatasets/articles/data.html].

## AttributeRank algorithm

### 1

**Table:**

1	#AttributeRank Algorithm ‐‐‐‐‐‐‐‐‐‐‐‐‐‐‐‐‐‐‐‐‐‐‐‐‐‐‐‐‐‐‐‐‐‐‐‐‐‐‐‐‐‐
2	# Requirement: This algorithm requires a data set to be normalised to the interval [0,1] before uploading for attribute ranking.
3	#‐‐‐‐‐‐‐‐‐‐‐‐‐‐‐‐‐‐‐‐‐‐‐‐‐‐‐‐‐‐‐‐‐‐‐‐‐‐‐‐‐‐‐‐‐‐‐‐‐‐‐‐‐‐‐‐‐‐‐‐‐‐‐‐‐‐
	**# Module One**
4	#This module converts all input values to binary using the threshold, 0.4
5	#‐‐‐‐‐‐‐‐‐‐‐‐‐‐‐‐‐‐‐‐‐‐‐‐‐‐‐‐‐‐‐‐‐‐‐‐‐‐‐‐‐‐‐‐‐‐‐‐‐‐‐‐‐‐‐‐‐‐‐‐‐‐‐‐‐‐
6	# Declare variables
7	DECLARE *i*, *j*, *k* AS INTEGER //Programme counters
8	DECLARE Data = ARRAY[1…*m*][1…*n*] AS INTEGER //2‐dimensional array
9	FOR *j* = 1 TO *n* //count columns
10	FOR i = 1 TO *m* //Count rows
11	IF Data[*i*,*j*] <0.4 THEN
12	Data[*i*,*j*] = 0 //Convert values less than 0.4 to zero
13	ELSE Data[*i*,*j*] = 1 //Convert values to one
14	END IF
15	NEXT *i*
16	NEXT *j*
17	#‐‐‐‐‐‐‐‐‐‐‐‐‐‐‐‐‐‐‐‐‐‐‐‐‐‐‐‐‐‐‐‐‐‐‐‐‐‐‐‐‐‐‐‐‐‐‐‐‐‐‐‐‐‐‐‐‐‐‐‐‐‐‐‐‐
	**# Module Two**
18	#This module evaluates the four quantities (*a* _11_ , *b* _10_ , *c* _01_ , and *d* _00_ ) required for the computation of risk difference
19	#‐‐‐‐‐‐‐‐‐‐‐‐‐‐‐‐‐‐‐‐‐‐‐‐‐‐‐‐‐‐‐‐‐‐‐‐‐‐‐‐‐‐‐‐‐‐‐‐‐‐‐‐‐‐‐‐‐‐‐‐‐‐‐‐‐
20	# Declare variables
21	DECLARE *a* _ *j* _ , *b* _ *j* _ , *c* _ *j* _ , *d* _ *j* _ As REAL //For *a* _11_ , *b* _10_ , *c* _01_ , *d* _00_ respectively.
22	DECLARE Outcome = ARRAY[1…*m*] AS INTEGER //1‐dim array for outcome variable
23	#
24	*a* _ *j* _ = 0; *b* _ *j* _ = 0; *c* _ *j* _ = 0; *d* _ *j* _ = 0 //Initialise sums for *a* _11_ , b_10_ , c_01_ , d_00_
25	# Separately sums up all occurrences of *a* _11_ , *b* _10_ , c_01_ , d_00_
26	FOR *j* = 1 TO *n* //column index position
27	FOR *i* = 1 TO *m* //row index position for independent variable
28	FOR *i* = 1 TO *m* //holds row index position for independent variables
29	FOR *k* = 1 TO *m* //row index position for outcome variable
30	IF *i* = *k* THEN //input and output index must be same
31	IF Data[*i*,*j*] = 1 AND Outcome[*i*,*j*] = 1 THEN
32	*a* _ *j* _ = *a* _ *j* _ + 1 //Counts *a* _11_
33	END IF
34	IF Data[*i*,*j*] = 1 AND Outcome[*i*,*j*] = 0 THEN
35	*b* _ *j* _ = *b* _ *j* _ + 1 //Counts *b* _10_
36	END IF
37	IF Data[*i*,*j*] = 0 AND Outcome[*i*,*j*] = 1 THEN
38	*c* _ *j* _ = *c* _ *j* _ + 1 //Counts *c* _01_
39	END IF
40	IF Data[*i*,*j*] = 0 AND Outcome[*i*,*j*] = 0 THEN
41	*d* _ *j* _ = *d* _ *j* _ + 1 //Counts *d* _00_
42	END IF
43	END IF
44	NEXT *k*
45	NEXT *i*
46	NEXT j
47	#‐‐‐‐‐‐‐‐‐‐‐‐‐‐‐‐‐‐‐‐‐‐‐‐‐‐‐‐‐‐‐‐‐‐‐‐‐‐‐‐‐‐‐‐‐‐‐‐‐‐‐‐‐‐‐‐‐‐‐‐‐‐‐‐‐
48	# **Module Three**
49	#This module computes risk difference for each independent variable as the attribute importance
50	#‐‐‐‐‐‐‐‐‐‐‐‐‐‐‐‐‐‐‐‐‐‐‐‐‐‐‐‐‐‐‐‐‐‐‐‐‐‐‐‐‐‐‐‐‐‐‐‐‐‐‐‐‐‐‐‐‐‐‐‐‐‐‐‐‐
51	# Declare variables
52	DECLARE exposureSum_ *j* _ , inexposureSum_ *j* _ AS REAL
53	DECLARE rateExposed_ *j* _ , rateInexposusd_ *j* _ , AttributeRank_ *j* _ AS REAL
54	#
55	WHILE *j* <= *n* DO
56	exposureSum_ *j* _ = a_j_ + b_j_
57	inexposureSum_ *j* _ = *c* _ *j* _ + *d* _ *j* _
58	rateExposed_ *j* _ = (*a* _ *j* _ /exposureSum_ *j* _ )
59	rateInexposed_ *j* _ = (*c* _ *j* _ /inexposureSum_ *j* _ )
60	AttributeRank_ *j* _ = |rateExposed_ *j* _ – rateInexposed_ *j* _ | //Absolute value
61	END WHILE
62	#
63	#The following prints variable names and their values of importance
64	#‐‐‐‐‐‐‐‐‐‐‐‐‐‐‐‐‐‐‐‐‐‐‐‐‐‐‐‐‐‐‐‐‐‐‐‐‐‐‐‐‐‐‐‐‐‐‐‐‐‐‐‐‐‐‐‐‐‐‐‐‐‐‐‐‐
65	PRINT variableName_ *j* _ + \TAB AttributeRank_ *j* _ + \ENTER
66	#‐‐‐‐‐‐‐‐‐‐‐‐‐‐‐‐‐‐‐‐‐‐‐‐‐‐‐‐‐‐‐‐‐‐‐‐‐‐‐‐‐‐‐‐‐‐‐‐‐‐‐‐‐‐‐‐‐‐‐‐‐‐‐‐‐
